# *In-planta* Sporulation Capacity Enhances Infectivity and Rhizospheric Competitiveness of *Frankia* Strains

**DOI:** 10.1264/jsme2.ME15090

**Published:** 2015-12-26

**Authors:** Laetitia Cotin-Galvan, Adrien C. Pozzi, Guillaume Schwob, Pascale Fournier, Maria P. Fernandez, Aude Herrera-Belaroussi

**Affiliations:** 1 PRES Université de Lyon, F-69361LyonFranceUniversité Lyon 1F-69622, VilleurbanneFrance; 2 CNRS, UMR 5557, Ecologie MicrobienneVilleurbanne, F-69622France

**Keywords:** *In-planta* sporulation, *Frankia*, competitiveness, infectivity, host-range, actinorhizal symbiosis

## Abstract

*Frankia* Sp+ strains maintain their ability to sporulate in symbiosis with actinorhizal plants, producing abundant sporangia inside host plant cells, in contrast to Sp− strains, which are unable to perform *in-planta* sporulation. We herein examined the role of *in-planta* sporulation in *Frankia* infectivity and competitiveness for root infection. Fifteen strains belonging to different Sp+ and Sp− phylogenetic lineages were inoculated on seedlings of *Alnus glutinosa* (Ag) and *A. incana* (Ai). Strain competitiveness was investigated by performing Sp−/Sp+ co-inoculations. Plant inoculations were standardized using crushed nodules obtained under laboratory-controlled conditions (same plant species, age, and environmental factors). Specific oligonucleotide primers were developed to identify *Frankia* Sp+ and/or Sp− strains in the resulting nodules. Single inoculation experiments showed that (i) infectivity by Sp+ strains was significantly greater than that by Sp− strains, (ii) genetically divergent Sp+ strains exhibited different infective abilities, and (iii) Sp+ and Sp− strains showed different host preferences according to the origin (host species) of the inocula. Co-inoculations of Sp+ and Sp− strains revealed the greater competitiveness of Sp+ strains (98.3 to 100% of Sp+ nodules, with up to 15.6% nodules containing both Sp+ and Sp− strains). The results of the present study highlight differences in Sp+/Sp− strain ecological behaviors and provide new insights to strengthen the obligate symbiont hypothesis for Sp+ strains.

*Frankia* is a soil filamentous actinobacterium that is capable of forming N_2_-fixing symbiotic root nodules on a diverse array of actinorhizal plants. Hyphae, diazovesicles (specialized cells for N_2_ fixation under aerobic conditions), and multilocular sporangia are the typical cell morphological features of *Frankia* grown in cultures (4, 28). Unlike hyphae and diazovesicles, sporangia are not constantly observed *in-planta*. Two types of nodules have been identified: Sp+ nodules, hosting abundant sporangia inside plant cells, and sporangia-free Sp− nodules (43). Sp+ nodules have been detected in 9 out of 23 actinorhizal plant genera, and at a high frequency in the genus *Alnus* (37). Historically, *in-planta* sporulation is considered to be a genetically stable characteristic (44), suggesting the existence of two types of *Frankia* strains (Sp+ *vs* Sp−). However, the inability to isolate strains from Sp+ nodules, despite numerous attempts, has limited their study.

To the best of our knowledge, Sp+ *Frankia* strains represent a unique model of endophytic symbionts with the ability to sporulate inside host cells. When sporulation occurs during plant infection, for example in the case of pathogenic fungi, spores are only produced on the surface of infected or necrotic tissues (40) and never inside living plant cells. Spores are dormant cells generally described as an optional adaptive strategy permitting spatial and temporal escape from local conditions that are unfavorable to growth (7, 25). In a symbiotic context, the ecological advantage of *in-planta* sporulation may be linked to the fate of numerous spores produced within Sp+ nodules once released into the soil. After nodule decay, spores provide abundant infective propagules, thereby increasing Sp+ strain infectivity and competitiveness in the soil environment (32). A limited number of studies, using host plant inoculations, have analyzed the behaviors of Sp+ and Sp− strains in the rhizosphere or during root infection, and suggested the higher infectivity of Sp+ (2, 13, 46). Sp+ *Frankia* strains may also differ in their host specificity (17, 23) and competitiveness for nodulation in co-inoculation assays (18).

However, these findings have been biased by the heterogeneity of the material used as inocula. Due to the non-cultivability of Sp+ strains, Sp− cultured cells have often been used as opposed to Sp+ crushed nodules in inoculation experiments, even though the phenolic compounds and tannins contained in nodule tissues are known to have an impact on symbiotic associations (20, 31). Furthermore, most of these studies were limited to Sp+ nodules from only one host origin (except in the large study by Markham *et al.* [23]) and, at that time, information was not available on the genetic diversity of Sp+ strains. A recent study conducted on *Alnus* field nodules from diverse geographical origins revealed at least two divergent lineages among Sp+ strains according to the host plant species: *A. incana* and *A. viridis* strains were grouped into clade 1 (1a and 1b, respectively), whereas *A. glutinosa* Sp+ strains were grouped into clade 5 (32). This genetic diversity suggests diversity in strain life history traits and, thus, is considered to have an impact on the ecological behavior of Sp+ strains. In addition, in the absence of molecular tools available to discriminate between Sp+ and Sp−, strain identification was based on microscopic observations of spores from hand-cut nodule sections. However, it was not possible to rule out false Sp− identification due to delays in the expression of the Sp+ phenotype, and the co-occurrence of both types of strains in the same nodule may have been missed in co-inoculation experiments (3).

Therefore, the aim of the present study was to re-examine the role of *Frankia in-planta* sporulation in (i) the infectivity and host range of *Frankia* strains and (ii) their competitiveness for root infection. Fifteen nodule sources of Sp+ and Sp− *Frankia* strains from three different *Alnus* species (*A. glutinosa*, *A. incana*, and *A. viridis*) were used, including representative strains from the different Sp+ phylogenetic lineages recently described (32). Sp+ and Sp− inocula were tested on *A. glutinosa* and *A. incana* host seedlings, and the resulting nodules were characterized at the molecular level using Sp+ and Sp− strain-specific oligonucleotide primers.

## Materials and Methods

### Plant culture

*A. glutinosa* and *A. incana* seeds from Lyon (Rhône, France) and Fond de France (Isère, France), respectively, were surface-sterilized by agitation for 30 min in absolute alcohol followed by 30 min in Ca(ClO)_2_ 3% (w/v) with 50 μL L^−1^ of Tween 20, and then rinsed three times in sterile water. Seeds were then incubated at room temperature on Fåhraeus medium (8) agar (10 g L^−1^) for germination. After 15 d, seedlings were transplanted into hydroponic pouches (8) containing 30 mL of Fåhraeus medium with 5 mM NH_4_Cl and incubated in a growth chamber (16-h d with 22°C day/18°C night temperatures).

### Selection of Sp+ and Sp− *Frankia* strains from field nodules

Since no pure-cultured Sp+ *Frankia* have been obtained to date, all *Frankia* strains were obtained from field nodules, including Sp− nodules, in order to ensure similar inoculation conditions for both types of strains. Nodules containing either Sp+ or Sp− *Frankia* were collected between September and October 2012 from different sites (previously described by Pozzi *et al.* [32]) and 3 different *Alnus* species: *A. glutinosa* (L.) Gaertn, *A. incana* (L.) Moench, and *A. alnobetula* (Ehrh.) K. Koch, hereafter referred to as *A. viridis* (Chaix) DC (Ag, Ai, and Av, respectively) (Table 1).

The nodule sporulation phenotype was determined (from at least one lobe) by microscopic observations of nodule hand sections stained with Lactophenol Blue (Réactifs RAL, Martillac, France), as previously described (32). Nodules were considered to have the Sp+ phenotype when more than one sporangium was observed in more than 50 infected plant cells. Otherwise, nodules were considered to have the Sp− phenotype. After surface sterilization of the nodules in calcium hypochlorite 3% (w/v) and peeling, nodular DNA was extracted and endophytic *Frankia* were genetically characterized by *pgk* and *dnaA* gene partial amplifications with specific primers (Table 2). Each PCR reaction contained in a final volume of 50 μL: 2 μL of DNA (0.1 μg mL^−1^), 1× PCR buffer, 0.2 mM of each dNTP, 2 mM MgCl_2_, 0.5 mM of each primer, 10% DMSO v/v, 16 μg of bovine serum albumin, and 2U Taq DNA polymerase (Invitrogen, Oxon, United Kingdom). The amplification conditions used were: initial denaturation at 94°C for 5 min, and 30 cycles including denaturation at 94°C for 1 min, annealing at 62°C for 1 min, and elongation at 72°C for 1 min. A final 10-min extension step was performed at 72°C. PCR products were purified using a MiniElute PCR purification Kit (Qiagen, Courtaboeuf, France) before sequencing (Biofidal, Villeurbanne, France) with an ABI 3730xl DNA Analyzer (Applied Biosystems, Foster City, California, USA). The resulting sequence alignments and pairwise-distance matrix calculations were performed using BioEdit software (version 7.2.2.). At least 5 lobes were genetically characterized for each nodule. Nodules for which all tested lobes resulted in *pgk* and *dnaA* sequences with more than 99.9% similarity were considered to contain a single *Frankia* strain. All nodules used as inocula in subsequent experiments met this criterion (data not shown). Seven Sp+ strains and 8 Sp− strains were used. Among the Sp+ strains, the two clades previously described (32) were represented with 2 Ag strains belonging to clade 5, and 2 Ai and 3 Av strains belonging to clade 1 (Table 1).

### Plant inoculation and growth assessments

Two distinct experiments were carried out in order to compare Sp+ and Sp− strain infectivity (experiment A) and competitiveness (experiment B).

#### Experiment A (Sp+/Sp− infectivity)

Two-month-old seedlings were transferred to nitrogen-free medium 24 h before inoculation. Each Sp+ and Sp− *Frankia* strain was inoculated on 17–42 seedlings per condition (Table 1) (total of 760 Ag and Ai seedlings), using a suspension of crushed nodules freshly collected in the field and stored at 4°C for a few days. The inocula density was used as described by Périnet *et al.* (29), with 2 g of nodules per one thousand seedlings. This relatively low density was selected in order to avoid the saturation of root infection sites, thereby allowing different nodulation rates to be highlighted. In each strain, field nodules were washed in 3% Ca(ClO)_2_ (w/v) with Tween 20 (50 μL mL^−1^), rinsed in sterile water, and then crushed with 3% (w/v) polyvinylpyrrolidone (PVP). Nodule suspensions were filtered through a 100-μm mesh filter (17). Plant roots were inoculated with 3 mL of crushed nodule suspensions directly in the hydroponic pouches and the seedlings were then incubated in a plant growth chamber for 5 months. Fåhraeus medium (8) was added weekly. In each plant, the total number of nodules was monitored every 2 weeks, and plant growth was followed by measuring the longest root and stem height.

#### Experiment B (Sp+/Sp− competitiveness)

Competitiveness experiments focused on Ag infective strains. The two Ag Sp+ strains, AgTrS1 and AgTyI.5, obtained from field nodules (Table 1) were confronted with two *in-vitro* cultured Sp− strains, ACN14a (28) and Mg60Ag2 (9). These two Sp− strains were selected because of (i) their genetic divergence with Ag Sp+ strains, allowing the design of specific oligonucleotides, and (ii) their different infectivities, with ACN14a being less infective than Mg60Ag2 on Ag. In order to standardize inocula for the competitiveness experiments (same age and plant origin of the crushed nodules), each of the four strains was inoculated on Ag seedlings grown in pouches. After approximately 4 months, the nodules produced were harvested and used to prepare crushed nodule suspensions. Four distinct Sp+/Sp− mixtures (AgTrS1/ACN14a, AgTrS1/Mg60Ag2, AgTyI.5/ACN14a, and AgTyI.5/Mg60Ag2) with 3 different ratios (v/v) were prepared: 15/85, 50/50, and 85/15 (percentage of Sp+ / Sp− inocula in the final volume), leading to 12 distinct conditions. A total of 15–16 plants per condition were inoculated using the previously described protocol. Four additional conditions based on single strain inoculations were included as controls. After 5 months in a growth chamber (the same conditions as those described for Experiment A), nodules were recorded and plant growth assessed as described above.

### Genotypic identification of *Frankia* strains present in nodules of inoculated plants

#### Experiment A (Sp+/Sp− infectivity)

Five months after inoculation, at least 2 nodules per plant were phenotyped and genotyped using *pgk* and *dna*A gene sequencing, as previously described, in order to ensure that the *Frankia* strains in the inocula were the same as those found in nodules.

#### Experiment B (Sp+/Sp− competitiveness)

Under each condition, at least 30 nodules randomly sampled from all plants were surface-sterilized and their DNA extracted as previously described (32). The following Sp+ and Sp− primers specific for *dnaA* were used: *F3 Sp+* and *R2 Sp+* primers for Sp+ strains, *F3 Sp*− and *R2 Sp*− primers for the ACN14a strain, and F2 and R2 primers for the Mg60Ag2 strain (Table 2). Nodular DNA was PCR amplified with Sp+ and Sp−-specific primers as described above. Amplicons were characterized by 2% w/v agarose gel electrophoresis.

### Statistical analyses

Statistical analyses were carried out using R software v. 3.0.1 (41).

#### Experiment A (Sp+/Sp− infectivity)

Chi-squared statistics with one degree of freedom were used to compare nodulated plant proportions, in order to test the effects of the strain-sporulating phenotype and host plant origin on the ability to infect Ag and Ai seedlings. The effects of possible interactions between these factors (in addition to the factor “species of inoculated seedlings”) on strain infectivity were tested with a Generalized Linear Model (GLM) on nodulated plant proportion data.

An ANOVA (Analysis Of Variance) followed by post hoc analyses (Tukey’s Honestly Significant Difference-HSD-tests) were used to test the effects of the strain phenotype and host plant origin on strain nodulation rates by comparing the log nodule number data (for normalized distribution) of infected plants.

Root length and stem height data were summed to calculate the “growth index” (cm) of nodulated plants. Since “growth index” data were not normally distributed, the effects of the strain-sporulating phenotype on plant development (growth index) was tested using Wilcoxon rank sum statistical tests.

#### Experiment B (Sp+/Sp− competitiveness)

Plant nodule numbers and growth indices were compared between the different conditions of co-inoculations and Sp+ controls (single Sp+ strain inoculations) in order to test the effects of the mixed strain co-inoculation on nodulation rate and plant development. An ANOVA followed by post hoc analyses (Tukey’s Honestly Significant Difference-HSD-tests) were used to compare the log nodule number data (for normalized distribution) of infected plants. Since growth index data were not normally distributed, Wilcoxon rank sum statistical tests were used.

## Results

### Infectivity of Sp+ and Sp− *Frankia* strains and plant growth

Seven Sp+ strains and 8 Sp− strains were inoculated on 456 Ag and 304 Ai seedlings. Three inocula never nodulated under the tested conditions (AvTol.2, AiGBh, and AvFF1.1). In all infective inocula, the first nodules appeared 2 weeks after inoculation and presented at least 3 lobes 60 days after inoculation. Phenotypic and genotypic characterizations always confirmed the presence of the *Frankia* strain used as the initial inoculum.

**Fig. 1:**
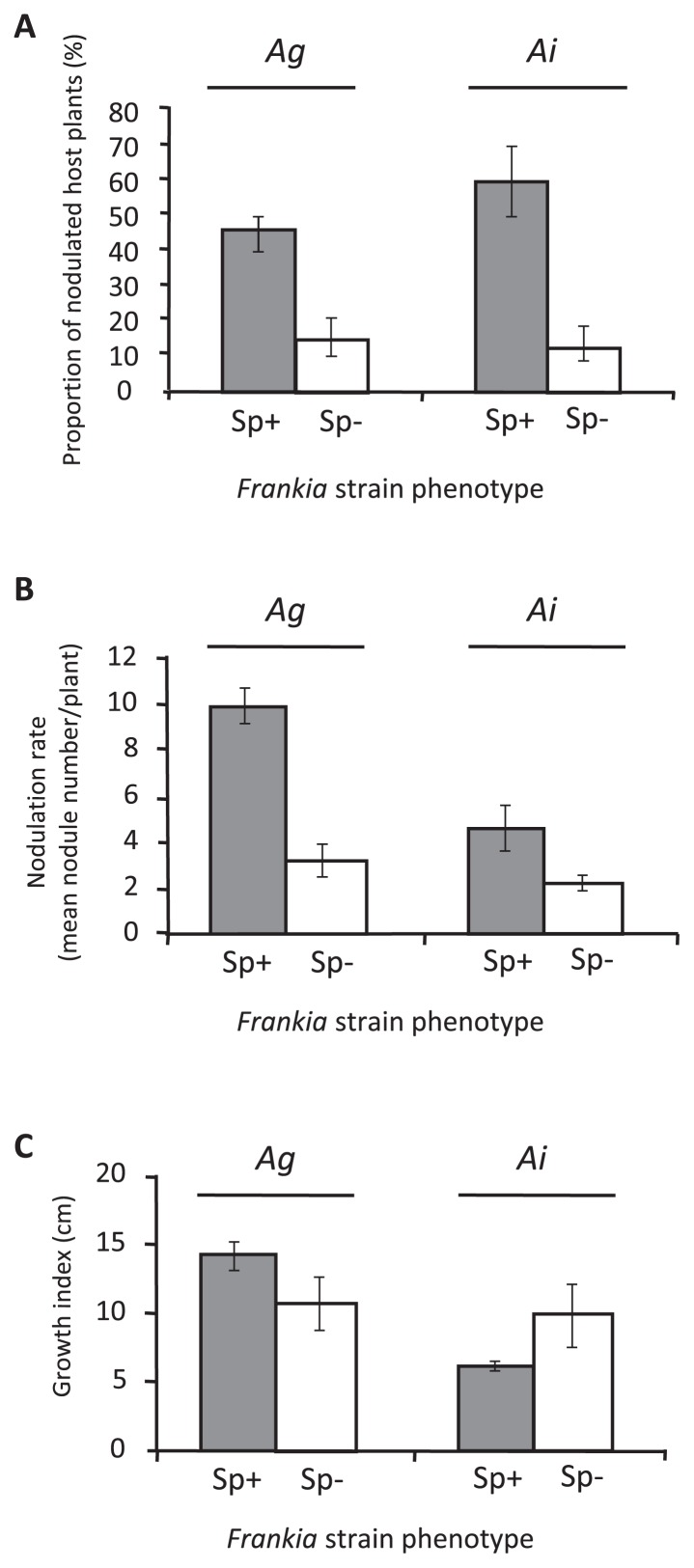
Effects of the *Frankia* strain-sporulating phenotype (Sp+ or Sp−) on the ability to infect *A. glutinosa* (Ag) and *A. incana* (Ai) seedlings (A), their nodulation rate (B), and plant growth (C) with “Growth index” (cm) corresponding to the sum between the root length and stem height data. Fig. B and C only include nodulated plants. Error bars indicate 95% confidence intervals (CI) computed with the modified Wald method (1) (A) or standard deviations (B and C).

The percentage of nodulated seedlings was significantly higher for Sp+ strains than for Sp− strains, on both Ag and Ai inoculated hosts (Fig. 1A). In total, 46.8% and 13.6% of Ag (Chi^2^ test, *p*-value=3.170e^−7^) and 60.6% and 10.5% of Ai (Chi^2^ test, *p*-value=1.350e^−13^) were infected by Sp+ and Sp− strains, respectively. The nodulation rate (mean nodule number per plant) similarly varied between Sp+ and Sp− strains (Fig. 1B). Sp+ strains produced significantly more nodules than Sp− strains on Ag seedlings (9.9±0.8 vs 3.2±0.9 nodules per plant, respectively, ANOVA, *p*-value=1.450e^−6^) and Ai seedlings (4.6±0.8 vs 2.2±0.3 nodules per plant, respectively, ANOVA, *p*-value=3.360e^−3^). Av strains presented different behaviors according to the inoculated host plant species (Table 3). While Av strains followed the general scheme on Ai (higher proportion of nodulated seedlings for Sp+ than for Sp− strains), they were weakly infective on Ag, irrespective of their phenotype (less than 6% of nodulated plants).

Multifactorial statistical analyses showed the significant effects of the interactions between strain phenotype, strain origin, and inoculated host species on strain infectivity expressed as the number of nodulated seedlings (GLM, *p*-value=0.007) and nodule number per plant (ANOVA, *p*-value=0.007).

The Sp+ strains chosen belonged to two divergent phylogenetic clades correlated to the host plant species: clade 1 (Ai and Av strains) and clade 5 (Ag strains) (Table 1) (32). The infectivity of clade 5 strains was significantly greater than that of clade 1 strains with a higher proportion of nodulated seedlings (91.5% vs 36.2% respectively, Chi^2^ test, *p*-value= 2.200e^−16^) and higher nodulation rate (10.7±0.8 vs 4.1±0.3 nodules per plant, respectively, ANOVA, *p*-value =1.060e^−13^).

As expected, Sp+ strains induced a lower plant index on Ai seedlings than Sp− strains (6.1 cm±0.3 and 9.9 cm±2.2, respectively—Wilcoxon rank sum tests, W=981, *p*-value= 0.024) (Fig. 1C). Conversely, no significant difference was observed on Ag seedlings (14.2 cm±0.9 and 10.7 cm±1.7, respectively—Wilcoxon rank sum tests, W=1309.5, *p*-value= 0.134). Depending on the strain origin (Ag, Ai, or Av nodules), Sp+ and Sp− inocula had different impacts on plant growth (Table 3): Ag Sp+ strains induced significantly more growth on Ag seedlings (Pairwise Wilcoxon rank sum test, *p*-value=6.800e^−4^) than on Ai seedlings (Pairwise Wilcoxon rank sum test, *p*-value>0.05). Ai Sp+ strains induced significantly less growth on Ai seedlings than Ai Sp− strains (Pairwise Wilcoxon rank sum test, *p*-value=1.910e^−2^), and similar results were observed on Ag seedlings (Pairwise Wilcoxon rank sum test, *p*-value>0.05).

### Host compatibility of Sp+ and Sp− *Frankia* strains

Identical host compatibility was observed for Sp+ and Sp− strains, with both being able to infect Ag and Ai seedlings (Table 3). Among the Sp+ strains tested, host preferences were observed according to their genotype. Clade 5 Sp+ strains infected significantly more Ag than Ai seedlings (96.3% vs 84.0%, respectively—Chi^2^ test, *p*-value=0.034), whereas clade 1 Sp+ strains infected significantly more Ai than Ag seedlings (51.5% vs 25.4%, respectively—Chi^2^ test, *p*-value=3.61e^−6^). In contrast, none of the Sp− strains showed significant differences in infected plant proportions between Ag and Ai seedlings (Chi^2^ test, *p*-value>0.05), although the same results were observed (different host preferences of Ag, Ai, and Av Sp− strains).

### Competitiveness of Sp+ and Sp− co-inoculated *Frankia* strains

Two Sp+ strains (AgTyI.5 and AgTrS1) and two Sp− strains (ACN14a and Mg60Ag2) were used to assess their competitiveness for Ag infection under co-inoculation conditions. Under single inoculation conditions (control plants), the Sp− strain Mg60Ag2 (9.8±1.4 nodules per plant) and the two Sp+ strains had similar nodulation rates (Tukey’s HSD tests, *p*-value>0.05). As expected, the ACN14a strain induced significantly fewer nodules than the 3 other strains (3.5±0.9 versus 10.7±0.9 nodules per plant, Tukey’s HSD tests, *p*-value<0.05).

The *Frankia* strain(s) present in nodules were identified using *dnaA* sequences. Irrespective of the strain used and the Sp+/Sp− ratio in the inocula, Sp+ strains were present in 98.3 to 100% of the nodules (Table 4). Under 5 of the tested conditions, Sp+ strains were found to co-exist with the Sp− strain in the same nodule (3.3 to 15.8% of the nodules). Sp− strains never recovered alone, with the exception of the 15/85 AgTyI.5/Mg60Ag2 inoculum, in which 1.7% of the nodules were Sp−. ACN14a, the low infective Sp− strain, was totally absent (100% of Sp+ nodules) in 5 out of the 6 co-inoculation tested conditions.

In both Sp+ strains tested, the number of nodules per plant slightly decreased with lower proportions in the inocula (Table 4). However, only the AgTyI.5/ACN14a co-inoculation with the ratio 15/85 was significantly different from the control (the AgTyI.5 single inoculation) (Tukey’s HSD test, *p*-value=4.464e^−3^).

**Fig. 2:**
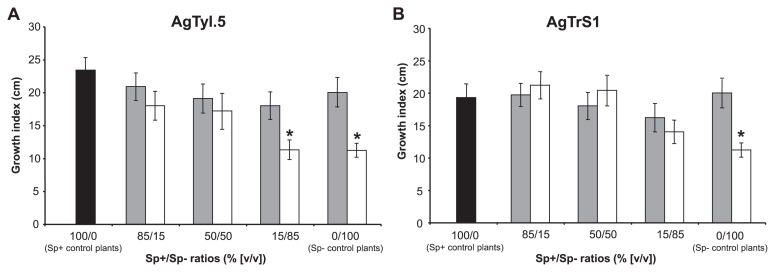
Effects of Sp+/Sp− *Frankia* strain co-inoculation on plant development. Two Sp+ strains were tested: AgTyI.5 (A) and AgTrS1 (B), in co-inoculations with two Sp− strains (Mg60Ag2 = grey bars and ACN14a = white bars). “Growth index” (cm) was the sum between the root length and stem height data. Error bars indicate 95% confidence intervals (CI). Stars indicate significant differences from the Sp+ control plants tested with Wilcoxon rank sum statistical tests (*p*-value<0.05).

No significant effect of the Sp+/Sp− co-inoculation was noted on plant growth, except for the 15/85 AgTyI.5/ACN14a and 100% ACN14a inocula (Sp− control plants), in which growth was significantly less than that in Sp+ control plants (Wilcoxon rank sum tests, *p*-value<0.05) (Fig. 2).

## Discussion

Sp+ *Frankia* strains represent a unique model of *in-planta* sporulation. Since bacterial sporulation commonly occurs in order to ensure survival under unfavorable growth conditions, its expression is paradoxical in a symbiotic context in which the nodule supplies a highly favorable ecological niche to the bacteria. Therefore, the role of the numerous spores produced in plant tissues is questionable. They may have an important ecological function in nodule decay, allowing new root infections once released into the rhizosphere. Decaying nodules may provide a source of infective particles depending on the survival and dispersal abilities of the bacteria released in the soil, as well as their competitiveness with other symbiotic strains. We herein attempted to examine Sp+ and Sp− strain behaviors in the rhizosphere by comparing their infectivity and competitiveness under co-inoculation conditions. In order to reflect the fate of decayed nodules in soil, exclusively crushed nodules were used as inocula. Since a large part of the released particles may be carried deep into the soil, with only small amounts reaching the roots, low density inocula were used.

Our results showed that Sp+ strains had (i) greater infectivity on Ag and Ai species (larger proportion of nodulated seedlings and higher nodulation rate—Fig. 1A and B) and (ii) better competitiveness than Sp− strains (Table 4). These two ecological traits are linked because the specific infectivities of two different strains are expected to condition the relative number of nodules they produce when simultaneously inoculated (26). The difference in infectivity and competitiveness between Sp+ and Sp− nodules may have been due to the higher number of infective particles released by Sp+ nodules. While one Sp− nodule releases only hyphae and vesicles in soils, one Sp+ nodule has the ability to also release a large number of spores. Based on microscopic observations of one thousand Sp+ nodule sections, we estimated that between 10^7^ and 10^8^ spores were released from 1 g of nodules (data not shown). The capacity of these spores to become infective propagules is supported by (i) their ability to germinate and form hyphae when transferred to fresh growth medium (16, 42), and (ii) the durability of nodulation capacity of old inocula (likely due to spore survival during culture senescence) (19). Moreover, isolated spores from cultured *Frankia* were shown to develop higher infectivity than hyphae (5). Spores released by Sp+ nodule tissues may completely invade the vicinity of the roots. Germination may occur in the soil in response to root secondary compounds, producing infective hyphae that may, in turn, induce new infections (16, 22, 42, 49). Alternatively, spores may also directly attach to the roots, as described for many species of sporulating biotrophic fungal pathogens (21, 48). The capacity of spores to rapidly saturate the root system, a limited resource, and, thus, deprive the Sp− strain of available infection sites, may result in competitive exclusion (10, 34) and the elimination of Sp− strains. Our results suggest that this occurs for low infectivity Sp− strains (*e.g.* ACN14a) (Table 4). Furthermore, the exclusion of Sp− strains by Sp+ strains may be the result of antagonistic interactions between both types of strains. *Actinobacteria* are known for their capacity to synthesize antibiotics and diverse secondary metabolites involved in antagonistic interactions with other microorganisms. Both competition types (competition for host infection sites or direct antagonism) have previously been described within symbiotic microbial populations (6, 14, 15, 26), and the combination of both cannot be excluded between Sp+ and Sp− strains. However, the non-cultivability of Sp+ strains renders these competition hypotheses hard to test in the case of actinorhizal symbioses.

The infectivity and competitiveness of rhizospheric bacteria may be modulated by the host plant. In the present study, no significant differences were observed in host compatibility between Sp+ and Sp− strains. Despite important variations in strain infectivity according to the inoculated plant species, both types of strains had the ability to infect Ag and Ai species. However, unlike Sp− strains, Sp+ *Frankia* strains showed differences in their ability to infect both host species. The infectivity of clade 5 (Ag) strains on Ag seedlings was significantly greater than that of clade 1 (Ai and Av) strains, whereas the infectivity of clade 1 strains was significantly greater on Ai seedlings than on Ag seedlings, confirming preliminary results (47) (Table 3). Therefore, Sp+ strains had better compatibility than Sp− strains when inoculated on their original host species, suggesting closer symbiotic relationships. Based on this hypothesis, some *Alnus* species may preferentially select Sp+ genotypes, as suggested by the higher occurrence of Sp+ strains in Av and Ai alder stands than in Ag alder stands (32). Symbiotic host plants are able to select their microbial partner; defense mechanisms are used during infection to regulate the root infection rate by a given strain (24, 27, 33). In the case of actinorhizal plants, the differential expression of such mechanisms has been reported based on metabolomic or transcriptomic approaches during the early infection steps, depending on the compatibility of the strains (11, 30, 31). Therefore, strain infectivity and competitiveness depend not only on the strain phenotype, but also additional factors, such as host-symbiont specificity, which strongly influence the success of infection.

The significant differences observed in infectivity and competitiveness between Sp+ and Sp− strains may have an impact on plant growth. Previous studies reported that Sp+ strains were less effective (plant dry matter, total N, and/or N-fixed) than Sp− strains (17, 38). By coincidence, both studies mainly based their conclusions on Ai Sp+ nodules and Sp− isolates from various hosts as inocula, tested on Ai host plants. Our results with Ai strains confirmed this conclusion (Fig. 1C). However, by testing different Sp+ and Sp− nodule origins from 3 different *Alnus* species, we unexpectedly found that Sp+ strains showed higher effectivity than Sp− (*e.g.* Ag strains on Ag seedlings). Thus, Sp+ and Sp− strains display different host specificities, which impacts not only on their infectivity and competitiveness for root infection, but also their effectivity. Moreover, by including nodules from several geographical origins, we noted a potential site effect on strain behavior (data not shown). Therefore, the hypothesis of Sp+ strains having lower efficiency cannot be fully verified unless numerous host and geographical origins are tested.

Our co-inoculation experiments demonstrated for the first time, using DNA-based genotype characterization, that two distinct *Frankia* strains inhabit the same nodular lobe (up to 15% of Sp+/Sp− co-inoculations—Table 4). Previous studies reported the presence of different *Frankia* strains in a single *Alnus* nodule (3, 39). The co-existence of both Sp+ and Sp− strains within a unique lobe may explain Sp− strain isolation from Sp+ typed nodules. Repeated culturing attempts from Sp+ nodules mostly failed, but led, in rare cases, to the isolation of *Frankia* strains. However, these cultures never differentiated *in-planta* sporangia when inoculated on different host species, and were, thus, considered to be Sp− strains (4, 35, 36). Although our protocol aimed at eliminating nodule surface contaminations, we cannot exclude the possibility that some external *Frankia* cells or their DNA remained and were amplified. Sp+ and Sp− strain localization and their relative proportions inside the nodule warrant further investigations. Additional molecular experiments based on *in-situ* hybridization from nodule sections and quantitative-PCR will shed light on those recurrent questions.

In conclusion, we herein confirmed the higher infectivity and competitiveness of Sp+ strains than Sp− strains as well as their higher host specificity. Moreover, these traits were differentially expressed depending on the phylogenetic clades that the Sp+ strains belong to. These results, associated with the non-cultivability of Sp+ strains, suggest they are dependent on the host plant for a large part of their life cycle and support the obligate symbiont scenario previously discussed (32, 37). Their higher infectivity and competitiveness may explain why they have been found to be highly invasive in the field (5, 32, 37, 45). The frequency at which Sp+ strains is detected is higher in old alder stands (12, 46), suggesting they may represent the final stage in the succession of *Frankia* populations (32). To date, *Frankia* spore persistence in the soil still remains uncharacterized, and further investigations on Sp+ strain detection directly from soil samples are needed in order to shed light on fundamental issues regarding their fitness during their saprophytic life or their fate in soils devoid of host plants.

## Figures and Tables

**Table 1:** Sp+ and Sp− field nodules used as inocula

Nodule phenotype^1^	*Alnus* species	Site designation^2^	Nodule acronym	No. of inoculated seedlings^3^	

				*A. glutinosa*	*A. incana*
Sp+	*A. glutinosa*	Thury	AgTyI.5	40	25
		Le Tremblay, Le-Bourget-du-Lac	AgTrS1	40	25
	*A. incana*	Ornon	AiOR8	40	24
		Allemont	AiAll	42	27
	*A. viridis*	La Bérarde	AvBI.5	40	25
		Col de la Croix de Fer	AvCf11.1	23	27
		La Toussuire	AvToI.2	40	27

Sp−	*A. glutinosa*	Arandon	AgARaG1	25	25
		Le Blanchet, Bourget-en-Huile	AgLB4.3	26	25
		Le Grand-Lemps	AgGL1	22	NT
	*A. incana*	Fond-de-France	AiFF2.1	25	24
		Le Blanchet, Bourget-en-Huile	AiGBh	17	NT
	*A. viridis*	Col de la Croix de Fer	AvCf3	22	NT
		Col de la Croix de Fer	AvCf13.1	26	24
		Fond-de-France	AvFF1.1	28	26

1 Sp+ and Sp− = *in-planta* sporulating and non-sporulating phenotypes, respectively.

2 All sites from Pozzi *et al.* (32).

3 NT = non-tested conditions.

**Table 2:** List of PCR primers used for *Frankia* strain genotyping

Gene	Primer name^1^	Sequence (5′→3′)	Tm (°C)
Field nodule identification (infectivity experiments)			
*dnaA*	dnAfdT7F	TAATACGACTCACTATAGGGGAGGARTTCACCAACGACTTCAT	62
	dnArvT3R	ATTAACCCTCACTAAAGGGACRGAAGTGCTGGCCGATCTT	
*pgk*	pgkFwdT3	ATTAACCCTCACTAAAGGGATGAGGACGATCGACGACCTGC	62
	pgkRevT7	TAATACGACTCACTATAGGGCGCSAGGAAGGTGAAGCACAT	

Sp+/Sp− discrimination in nodules (co-inoculation experiments)			
*dnaA*	F2 Sp−	CCATGGAGACGCCGAAGTAC (*1341*)^2^	65
	F3 Sp−	CGTCCGGGATCAGGTCG (*1275*)	64–65
	R2 Sp−	CATCGCGATCCTGTCGAAGAAG (*1089*)	65
	F3 Sp+	GCGTCAGGGATCAGGTCA (*1275*)	64
	R2 Sp+	CATCGCGATCCTGTCGAAAAAA (*1089*)	64

1 “fd”, “Fwd”, or “F” in the name indicate forward primers and “rv”, “Rev”, or “R” indicate reverse primers.

2 Position on the *dnaA* sequence of the ACN14a strain (GenBank accession NC_008278).

**Table 3:** Sp+ and Sp− strain infectivities on *Alnus glutinosa* and *A. incana* seedlings.

Inoculated strains		% of nodulated plants^1^		Nodule mean number per plant^2^		Plant growth index (cm)^2^	
			
Phenotype	Original *Alnus* species	*A. glutinosa*	*A. incana*	*A. glutinosa*	*A. incana*	*A. glutinosa*	*A. incana*
Sp+	*A. glutinosa*	96.3 (80)	84.0 (50)	13.7±0.8	5.2±0.5	18.7±1.1	6.3±0.6
	*A. incana*	52.4 (82)	82.4 (51)	7.0±0.0	4.9±0.4	6.6±0.4	4.5±0.3
	*A. viridis*	3.9 (103)	31.6 (79)	2.3±0.6	3.3±0.7	10.8±3.3	8.4±0.5

Sp−	*A. glutinosa*	27.4 (73)	16.0 (50)	3.0±0.9	1.9±0.4	8.1±1.2	6.8±1.2
	*A. incana*	4.8 (42)	16.7 (24)	4.1±0.5	3.0±1.2	30.7±1.7	17.5±5.2
	*A. viridis*	5.3 (76)	2.0 (50)	1.5±0.5	1.0 (1 plant)	13.7±4.9	5.0 (1 plant)

1 (n) number of inoculated seedlings.

2 only nodulated plants are included.

**Table 4:** Competitiveness of co-inoculated Sp+ and Sp− strains on *Alnus glutinosa* host plants.

Inoculated strains		Sp+ / Sp− ratio	Nb nodule per plant	Nodule proportions (%)^1^		
	
Sp+	Sp−			Sp+	Sp−	Sp+/Sp−
AgTyI.5	None (Sp+ control)	100/0	10.0±1.3	100.0	0.0	0.0

	Mg60Ag2	85/15	8.2±1.0	96.7	0.0	3.3
		50/50	6.9±1.0	84.2	0.0	15.8
		15/85	5.9±0.6	98.3	1.7	0.0

	ACN14a	85/15	7.5±1.9	100.0	0.0	0.0
		50/50	7.4±2.2	96.6	0.0	3.4
		15/85	4.3 *±0.8	100.0	0.0	0.0

AgTrS1	None (Sp+ control)	100/0	12.3±2.1	100.0	0.0	0.0

	Mg60Ag2	85/15	10.2±1.3	96.7	0.0	3.3
		50/50	8.9±1.9	100.0	0.0	0.0
		15/85	6.7±1.1	96.7	0.0	3.3

	ACN14a	85/15	12.5±1.9	100.0	0.0	0.0
		50/50	9.0±1.8	100.0	0.0	0.0
		15/85	6.2±1.2	100.0	0.0	0.0

1 Sp+: % of nodules with strain AgTyI.5 or AgTrS1; Sp−: % of nodules with strain Mg60Ag2 or ACN14a; Sp+/Sp−: both Sp+ and Sp− co-existing strains.

* Significantly different from the corresponding Sp+ control plants (without Sp− co-inoculation, grey lines), using Tukey’s HSD tests (*p-value*<0.05).
